# Inclusion in and through disability sport? A scoping review using the examples of goalball and wheelchair basketball

**DOI:** 10.1016/j.jsampl.2025.100096

**Published:** 2025-03-25

**Authors:** Felix Oldörp, Christopher Mihajlovic, Martin Giese

**Affiliations:** a Heidelberg University of Education, Faculty of Natural and Social Sciences, Sports Science & Sport Pedagogy, Im Neuenheimer Feld 720, 69120 Heidelberg, Germany; b Rohräckerschulzentrum Esslingen, SBBZ Geistige Entwicklung, Traifelbergstr.2, 73734 Esslingen am Neckar, Germany; c Philipps-Universität Marburg, Department of Education, Institute of Sport and Motology, Barfüßerstr. 1, 35032 Marburg, Germany

**Keywords:** Athletes, Disabled persons, Exercise, Participation, Physical education and training, Reverse integration

## Abstract

**Background:**

Inclusion in sport has been empirically investigated and demonstrated the potential for promoting inclusion. Nevertheless, a comprehensive overview of how inclusion is understood and theoretically conceptualized within the context of disability sports research is still lacking. Therefore, a scoping review has been conducted to map the existing literature concerning the conceptions of inclusion in disability sports using goalball and wheelchair basketball as examples.

**Methods:**

For the scoping review a comprehensive search of five databases was conducted, resulting in the identification of nine articles that were deemed eligible for review. Coding of the data was performed to categorize specific elements, aiming to identify key features and concepts related to inclusion.

**Results:**

All reviewed articles recognize sport as an environment in which people of all (dis)abilities can participate. Abilities acquired in and through sport were presented as an important element for inclusion. Inclusion concepts in the articles were based on different concepts. But the concepts were not always clearly defined. In the qualitative studies analyzed, the concepts of inclusion were expanded to include subjective feelings such as equality and belonging based on the statements of the participants.

**Conclusions:**

The findings highlight the need for further research using qualitative methodologies that extend beyond the spatial dimension of inclusion, encompassing other dimensions to provide a more comprehensive understanding of inclusion in (disability) sport. Since discussions about inclusion concepts have been primarily driven by physical education research, future research should also focus on recreational and popular sports to strengthen inclusive sports programs.

## 1 Introduction

The United Nations considers sports in its 2030 Agenda as an effective tool for the social inclusion of people with disabilities [1]. Article 30, Section 5 of the United Nations Convention on the Rights of Persons with Disabilities (CRPD) calls for the equal participation of people with disabilities in sports activities [2]. In general, research on sport participation and inclusion has been well reported in the literature [3,4] and has been intensively discussed and empirically investigated on various levels, from school sports to elite sports. The primary focus is on the field of education [4]. Nevertheless, inclusion is also discussed at various levels in recreational sport [5,6], as well as in competitive sport and within the Paralympic system [7]. However, there is an ongoing debate about the definition of inclusion, and still, the term remains vague [8], because different areas use different methods and concepts of inclusion [4]. “There are many more interpretations and permutations of inclusion in disability sport than could be encapsulated within the simple dichotomy of exclusion versus inclusion” [9, p. 188]. Holland et al. [10, p. 275] distinguish between two categories of inclusion: inclusion as “a physical space or placement […], a philosophy related to the socially constructed environment”. Goodwin and Peers [9] posit that the concept of inclusion can be understood in two different ways. Firstly, it can be conceptualised as the provision of diverse participation opportunities for individuals with disabilities within various sporting structures. Secondly, it can be viewed as an individual's personal experience. Haegele and Maher [11, p. 386] speak of “inclusion as intersubjective experiences”. Those approaches explicitly want to make the voices of people with disabilities heard [12] and work with an intersubjective understanding of inclusion. These approaches primarily ask whether people with disabilities feel included in their respective settings and spaces [11].

### 1.1 Promoting the inclusion of people with disabilities through sport

One of the International Paralympic Committee's (IPC) objectives in their Strategic Plan 2023–2026 is “advancing disability inclusion” [13, p. 7]. This objective is compatible with the goal of the Special Olympics “to help persons with intellectual disabilities participate as productive and respected members of society by offering them a fair opportunity to develop and demonstrate their skills and talents through sports training and competition and by increasing the public's awareness of their capabilities and needs” [14, 1.02]. The IPC and Special Olympics (as well as the International Committee of Sports for the Deaf (ICSD)) are organizations that offer competitions in disability sports. Disability sport^1^ refers to sport that has been designed or adapted for athletes with disabilities or is practiced by people with disabilities [9,15]. In disability sport, people who are diagnosed with certain impairments usually practice athletic activities that are separated from their able-bodied peers and, in many cases, their peers with different types of impairment due to a classification system [9,16]. On one hand, because of these practices of segregation, several scholars have criticized the Paralympics and Special Olympics games – against their self-claim – to be not inclusive due to the selective classification criteria with norm-based ability expectations [17,18]. On the other hand, research has shown that disability sports may give athletes with disabilities a stage to demonstrate their ability and breaking down preconceptions [16], while promoting a sense of empowerment and social inclusion among participants [19,20].

Inclusion in and through disability sport has been the subject of extensive research in the past. However, as described above, definitions of inclusion vary and often focus on school sport. But as Kiuppis [4, p. 5] points out “when the word ‘inclusion’ is used in the context of sport, do we actually associate the same theories, concepts and methods as in Inclusive Education?” In front of this backdrop, it becomes evident that it is important to understand how inclusion in disability sports research is understood and operationalized by researchers in their respective work. Addressing this research gap, the present scoping literature review aims to investigate how inclusion is conceptualized in disability sport research using the sports of goalball and wheelchair basketball as examples.

### 1.2 Goalball and wheelchair basketball in the context of inclusion

The examples of goalball and wheelchair basketball were chosen because the two sports have been attributed a great potential for fostering inclusion. Wheelchair basketball “is an example of a recognized sport where people with an impairment are acknowledged as athletes first rather than people with disability” [21, p. 26]. This might have an impact on the perception of the sport as a whole, questioning the term disability sport [22]. Studies that examined participants without disabilities playing in disability sports, showed that the participants without disabilities questioned their own abilities or reveal a permanent “border crossing between ability and disability” [23, p. 30] and therefore challenge ableist assumptions. According to studies, reverse integration can contribute to inclusion by enabling people with and without disabilities to share common experiences in sport [21,24] or by providing sport opportunities [25]. Reverse integration refers to the inclusion of athletes without disabilities in a sport that has traditionally been designed for athletes with disabilities [15]. At a reverse integration context, people with disabilities are in the majority and people without disabilities are in the minority [26]. One area where reverse integration does not exist is at the Paralympic Games, as participation in this event is reserved for athletes with a (physical) disability only. Against this backdrop, the significance of reverse integration for disability sports and inclusion is controversially discussed in the literature (see, among others [[26], [27], [28]]).

Wheelchair basketball is a sport with a long history in reverse integration [26,27] and was first played by war veterans in the USA in 1945. Independently, British war veterans began playing a similar game in 1948. In 1955 both countries competed against each other, leading to the transition from wheelchair netball to wheelchair basketball. It was one of the sports included in the inaugural Paralympic Games, which were held in Rome in 1960 [29].

There have also been some recent developments in the area of reverse integration in goalball [23]. Goalball is a game that has been developed for individuals with visual impairments. It was invented in 1946 by Austrian Hans Lorenzen and German Sepp Reindle to aid the rehabilitation of blinded war veterans. It is a sport that has not been adapted from familiar sports but has been newly developed. To ensure equal opportunities, all goalball players wear eyeshades. The game has been part of the Paralympic Games since 1976 [30].

In recent reviews where researchers have been concerned with goalball, the focus was on fitness tests [31], performance parameters [32], or game analysis [33] while in the context of wheelchair sports, the focus is placed on Paralympic elite sports [34], technologies, mobility, and performance tests [35,36]. To our knowledge, no systematic summary concerning the conceptions of inclusion in goalball and wheelchair basketball exists in the literature. Therefore, we conducted a scoping review to map the existing academic literature concerning the conceptions of inclusion in disability sports using the examples of goalball and wheelchair basketball.

## 2 Methods

A scoping review was chosen because it is an effective method for mapping literature, clarifying key concepts, and identifying potential gaps in a specific research area [37]. Taking our theoretical considerations in regards to inclusion into account, the overarching research question of this analysis is: How is inclusion conceptualized from a theoretical perspective by the authors in inclusion research using goalball and wheelchair basketball as examples?

In accordance with the main research question, the following sub-questions were examined: a) How do the authors evaluate the inclusive potential of goalball and wheelchair basketball? b) What understanding of inclusion do the athletes with disabilities have? c) What is the background of the participants that took part in the studies (gender, age, (dis-)abilities)?

To minimize bias and support transparency in the identification, selection, assessment and summary of studies, the PRISMA-ScR checklist (Preferred Reporting Items for Systematic Reviews and Meta-Analyses Extension for Scoping Reviews) was used [38].

### 2.1 Inclusion and exclusion criteria

In order to capture all relevant results in the screening process, SPIDER (Sample, Phenomenon of Interest, Design, Evaluation, Research Type) was used as a search strategy tool. As an alternative to PICOS (Population, Intervention, Comparison, Outcome, Study Design), SPIDER is suitable for scoping reviews as well as for qualitative research and sports science inquiries [[39], [40], [41]]. In this study, the components of the SPIDER framework were defined as shown in Table 1.

**Table 1:** Components of the SPIDER framework.

Component	Description
Sample (S)	Adults with disabilities who play goalball or wheelchair basketball
Phenomenon of Interest (PI)	Inclusion or reverse integration in the sports of goalball and wheelchair basketball
Design (D)	Qualitative studies, quantitative studies, mixed-methods studies, reviews, reports, essay.
Evaluation (E)	Definitions and descriptions of inclusion, results on inclusion experience, inclusive potential of the sport
Research type (R)	Qualitative research, quantitative research, mixed-methods research

The inclusion and exclusion criteria were defined according to the research questions. The criteria were formulated in advance by the first author and then checked for consistency by the second author. This was done to reduce personal bias [42]. This scoping review included academic articles in English and German language. The search was deliberately limited to articles in English and German, as the team of authors is familiar with both languages. This helped to facilitate the validation of the terms and concepts used in the studies and thus minimise interpretation difficulties. To obtain a comprehensive overview of the research field and to minimise the risk of a temporal bias, no publication period was specified for the articles. Published articles were included if they (a) addressed inclusion in goalball or wheelchair basketball, (b) examined programs in recreational, leisure, competitive, or rehabilitative sports, (c) involved adult goalball and wheelchair basketball players, and (d) were presented in the form of a journal article (with or without peer review). The duration of the athletes' participation in wheelchair basketball or goalball was not considered. The exclusion criteria pertained to published studies that (a) focused on children's and youth sports as well as school sports, (b) were written in a language other than English or German, (c) included people with and without disabilities but did not provide data separately for each cohort, (d) included children, adolescents and adults but did not separate data by age.

### 2.2 Search strategy

In April and May 2024, five databases relevant to sports science inquiries were screened: SportDiscus, Scopus, PubMed, Education Resources Information Center (ERIC), Bundesinstitut für Sportwissenschaft (BIsp) Sport Und Recherche im Fokus (SURF) (BIsp-SURF). Truncations were used in all searches to ensure that all relevant articles were captured. Since the search was limited to English and German articles, the search filter was restricted to displaying English and German articles as part of a drill-down strategy. The first author conducted the search, the initial screening, including the removal of duplicates, the title screening, and abstract screening. The second author independently reviewed the results after the title and abstract screening. For the full-text screening, the first author conducted the initial review, and the second author checked the decisions. Any discrepancies or disagreements during the process were discussed and resolved collaboratively to ensure consistency and reliability in the study selection. Table 2 lists the search terms and the results obtained for each database. Following the identification of all relevant articles via the selected databases, the screening process was conducted in three steps. First, search terms were applied to the titles to identify potentially relevant studies. Second, abstracts of these studies were screened manually to assess their alignment with the inclusion criteria. Finally, full texts of the remaining studies were reviewed to confirm their eligibility.

**Table 2:** Number of results per database.

Search terms:	“wheelchair basketball” AND inclusi∗ OR reverse integration NOT school Goalball AND inclusi∗ OR reverse integration NOT school Rollstuhlbasketball AND inklusi∗ OR reverse integration NOT Schule Goalball AND inklusi∗ OR reverse integration NOT Schule without language filter (with English/German language filter)				

**Database**	**SportDiscus**	**Scopus**	**PubMed**	**ERIC**	**BIsp-SURF**

Number of records goalball	19 (15)	4 (3)	17 (17)	0 (0)	4 (4)
Number of records wheelchair basketball	34 (31)	34 (29)	20 (20)	2 (2)	21 (19)

To find articles that may have been overlooked in the initial database search, a backward citation search was conducted by the first author. This involved reviewing the reference lists of studies included in the full-text screening for additional relevant articles. The citations of content-relevant sections in the articles' reference lists were sought and compared with the initial search results. Newly discovered titles were then subjected to full-text screening [43]. An assessment of the methodological quality of the articles included in the scoping reviews was not undertaken [37].

### 2.3 Data analysis

The aim of the scoping review was to capture inclusion concepts in inclusion research using goalball and wheelchair basketball as examples. For this reason, it was necessary to undertake a basic coding of the data into specific categories in order to determine the concepts and identify key features related to the concept of inclusion [37]. The data analysis was conducted based on content-related structuring of qualitative text analysis [44] in a multistep coding process with deductive-inductive category formation [44]. The main categories were developed by the first author and checked by the second author according to the theoretical framework of the article.

The main categories were formulated based on theoretical assumptions underpinned by previous research results and the guiding research questions. The understanding of the term ‘disability’ influences the conceptualization of inclusion and the design of physical activity programs [45]. Additionally, reverse integration may impact the conceptualization of (dis)ability [46] and inclusion [21,25]. The demonstration of abilities seems to be understood as a means of recognising people with disabilities in society and is thus intended to promote inclusion [47].

**Fig. 1:**
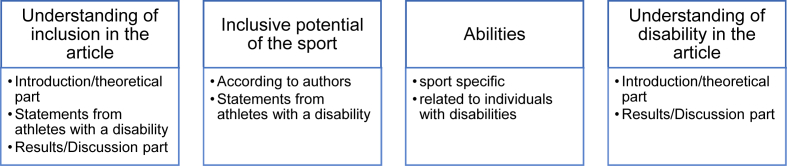
Differentiated category system with main and sub themes.

The first step consisted of coding the included articles according to the main categories, which was performed by the first author. Information related to the research questions was created from each article that met the inclusion criteria and organized into an article synthesis grid to thematically code the data in relation to the study's main questions. In the second step, the material was further analyzed by the first author, whereby the categories were inductively revised and differentiated. The themes were then reviewed together by the first and second author until consensus was reached. Similarities between results were identified as patterns and presented as results [44]. Fig. 1 presents the final category system with four main categories and the differentiated subcategories.

## 3 Results

### 3.1 Search results

**Fig. 2:**
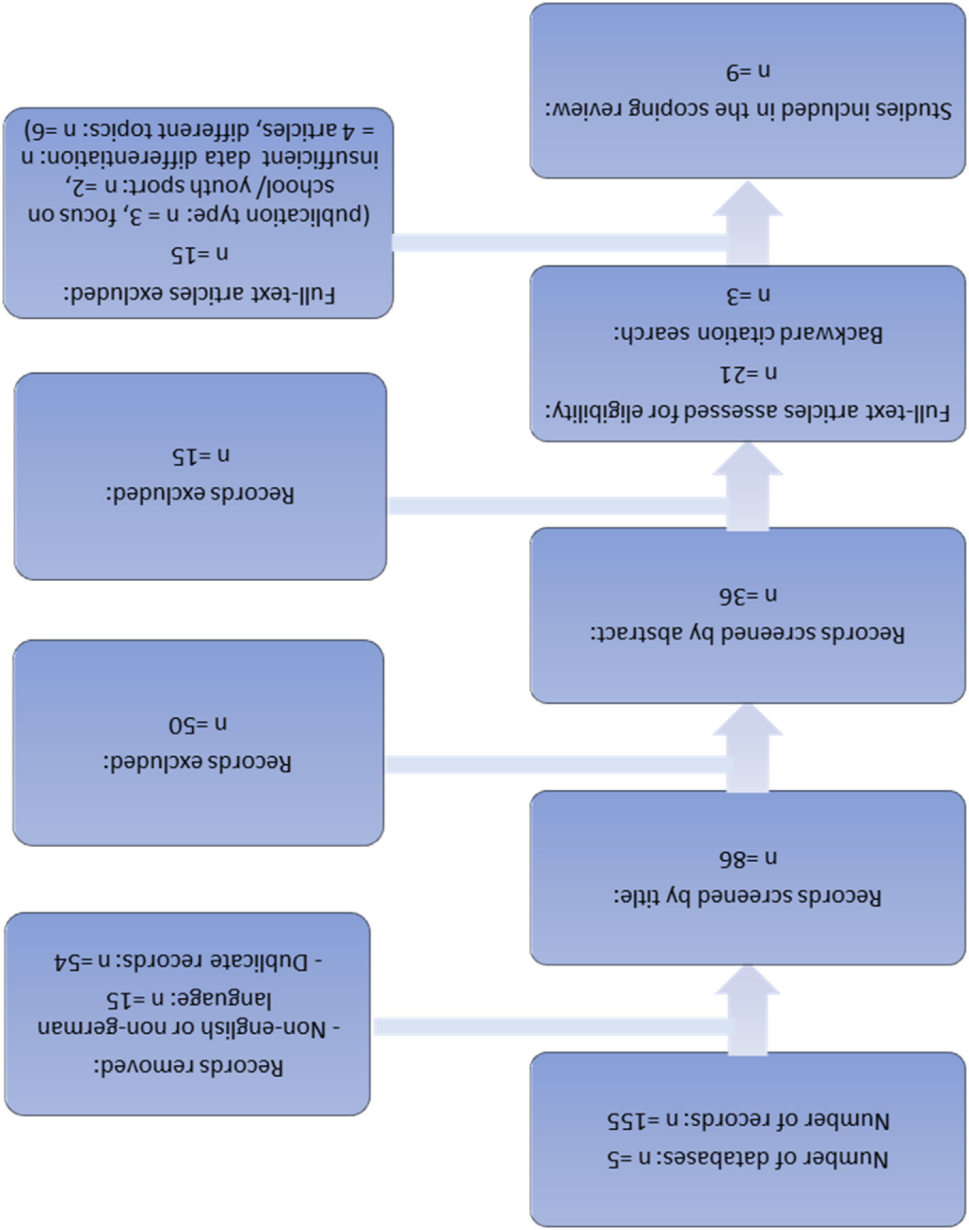
Screening process.

24 articles were included in the full-text screening and 15 articles were excluded after screening. The reasons for exclusion are included in Fig. 2. Finally, nine articles were included in the review process. Fig. 2 shows the number of studies after each screening step.

### 3.2 Study characteristics

Table 3 provides an overview of the nine articles included in the review process. Of the nine articles reviewed, eight were written in English and one in German [48]. Only one article [49] did not focus on the concept of reverse integration, one study investigated goalball [25]. In terms of methodology, five qualitative studies [22,25,46,49,50] and four theoretical contributions [27,28,48,51] were identified. The qualitative studies provided information from 41 individuals with and without disabilities, who were active in either wheelchair basketball or goalball. Of the 41 participants in total, 21 were female and 20 male. Two studies [22,49] exclusively interviewed individuals with disabilities. Of the 41 participants relevant to the analysis, 28 had a disability. The following section will present the significant findings.

**Table 3:** Characteristics of the included articles.

Author, year	Title	Sport	Reverse Integration	Country	Type of Study	Participants
Brasile, 1992	Inclusion: A developmental perspective. A rejoinder to “Examining the concept of reverse integration”	Wheelchair basketball	Yes	USA/CAN	Viewpoint	/
Brasile, 1990	Wheelchair sports: A new perspective on integration	Wheelchair basketball	Yes	USA/CAN	Viewpoint	/
Kampmeier, 2003	Als Fußgängerin beim Rollstuhl-Basketball. Eine etwas andere Inklusion (title translation: As a walking person in wheelchair basketball. A slightly different kind of inclusion)	Wheelchair basketball	Yes	GER	Report	/
Kayama et al., 2023	“The wheelchair really is just a piece of athletic equipment to play the sport of basketball”: The experience of college athletes with disabilities navigating social inclusion and exclusion	Wheelchair basketball	No	USA	Interview	7 male athletes with disabilities; 17–22 years
MacDonald et al., 2020	“You think differently after playing this sport”: Experiences of collegiate goalball players	Goalball	Yes	USA	Interview	6 athletes (18–47 years; 4 female, 2 male) including 2 with disabilities (1 female, 18 years, 1 male, 24 years)
Ramsden et al., 2023	Sport participation for people with disabilities: Exploring the potential of reverse integration and inclusion through wheelchair basketball	Wheelchair basketball	Yes	GB	Interview	11 athletes (18–41 years; 4 female, 7 male) including 7 with disabilities (gender and age unknown)
Spencer-Cavaliere & Peers, 2011	“What's the difference?” Women's wheelchair basketball, reverse integration, and the Question(ing) of disability	Wheelchair basketball	Yes	USA/CAN	Interview	9 female athletes with disabilities; 22–55 years
Stanojevic et al., 2023	The inclusive adaptive sport program on a college campus: Changing the narrative	Wheelchair basketball	Yes	USA	Interview	8 athletes (21–31 years; 4 female, 4 male) including 3 with disabilities (22–23 years; 2 male, 1 female)
Thiboutot; Smith & Labanowich, 1992	Examining the concept of reverse integration: A response to Brasile's “New perspective” on integration	Wheelchair basketball	Yes	USA	Viewpoint	/

### 3.3 Concepts of inclusion

All articles addressed inclusion in relation to the category of disability. The understanding of inclusion in all articles is underpinned by the joint participation of people with and without disabilities in a specific (often athletic) context. In eight out of nine articles, this was facilitated through reverse integration. Some authors [22,25,46,51] provided a concrete definition of reverse integration in their theoretical sections. Furthermore, the articles revealed a broad understanding of inclusion, which often remained vague, as beyond the definition of reverse integration, no specific description of inclusion was given in the theoretical part of the articles. Four conceptions of inclusion in the theoretical part formulated by the authors can be summarized as...

• ... accessibility [28].
• ... integration of people with disabilities into society [27,51].
• ... the opposite of exclusion [49,50] also [28].
• ... joint participation in sports (e.g., in terms of reverse integration) [22,25,46,48].

All qualitative studies were characterized by the fact that, in the results or discussion chapters, the concept of inclusion was expanded based on the participants' statements. In this process, the inclusion concepts were mainly extended by subjective feelings such as equality [25,49], value [22] and a sense of belonging [46,50].

### 3.4 Sport as a tool for inclusion

Generally, all articles regarded sports as an important means to enhance the inclusion of people with disabilities. A particular potential was attributed to reverse integration in wheelchair basketball and goalball. All authors (except [28]) acknowledged the significant inclusive potential of the respective sport through reverse integration, whereby Kampmeier [48, p. 48] referred to the need for rule adjustments in wheelchair basketball to prevent people with disabilities from being “pushed out of their sport” by those without disabilities. In summary, the authors identified the inclusive potential primarily based on the following aspects:

• Initiating interactions in sportive and non-sportive contexts [27,46,48],
• Improving skills and better understanding the abilities of people with disabilities [22,25,27,46,51],
• Raising awareness and changing attitudes towards people with disabilities [22,25,49,51].

Only few quotes from individuals with disabilities could be assigned to these aspects. From their perspective, the potential is also regarded positively, as reverse integration draws attention to their needs and injustices in daily life [27], allows everyone to participate, and fosters a sense of equality [50]. However, participants in the study by Spencer-Cavaliere and Peers [22] expressed the desire for reverse integration to remain excluded from the Paralympics.

A central finding regarding the inclusive potential in most articles is the transformation of the concept of disability in the results/discussion sections. Disability is regarded as a fluid category [22,46], which is in contrast to the rigid conception of disability in sports regulations underpinned by pre-conceptions about performance-related fairness.

### 3.5 The importance of abilities for inclusive processes

A majority of the reviewed articles emphasize that people with disabilities are confronted in their everyday lives with deficient assumptions about abilities of people without disabilities [22,25,49,51]. Some of the articles reviewed suggest that abilities should be considered an integral part of inclusion, and their development should be actively promoted. Brasile [27,51] emphasized that skills acquired through sport are essential for inclusion. These skills go beyond the athletic ability to participate in the specific sport and serve as a universal assessment standard, regardless of disability. Similarly, Ramsden et al. [46] emphasized the importance of recognizing and acknowledging the abilities of people with disabilities. In line with Stanojevic et al. [50] these authors argued that enhancing abilities through wheelchair basketball not only improves athletic performance but also has positive effects beyond sports. This can be seen, for example, in the fact that people with disabilities transfer the self-confidence and resilience they have acquired through sport to their academic studies [50]. However, the concept of abilities often remains unclear, despite its intention to replace disability as a criterion for evaluating individuals [27,46,51].

In the following, the implications of these results will be discussed in more detail in relation to our research questions, critically analyzing the significance and application of different concepts of inclusion. In addition, the role of (dis)abilities in the context of inclusion and the impact of ableism on the perception and participation of people with disabilities are discussed. Furthermore, research gaps will be identified, and conclusions from our analysis will be drawn.

## 4 Discussion

In this scoping review, existing research on inclusion in goalball and wheelchair basketball was summarized and analyzed. The research focused on how inclusion is understood and theoretically conceptualized using these two sports as examples. The results are based on nine articles that met the specified inclusion criteria. The analysis revealed that the reviewed articles were based on different concepts of inclusion that were not always clearly defined in the instruction/theoretical part of the article.

In the context of sports, inclusion often refers to spaces where athletes with disabilities or disability sports themselves are integrated [11,52]. In the articles included in our analysis, the main focus was frequently on spatial inclusion (whether athletic or societal). Haegele and Wilson [8], refer to this approach as the “inclusion-only placement approach,” and Connor and Berman [53], describe inclusion as “a technical response to change”. When the term refers to a space, it often remains unclear whether inclusive experiences actually occur within that space [54].

The emphasis on purely structural and organizational levels of inclusion tends to neglect the psychological dimension. The psychological dimension refers to the subjective feeling of a person being an important part of their environment. These feelings can arise from acceptance, belonging, participation, success, and appreciation [55,56]. Such experiences are subjectively shaped and can be available to individuals with disabilities in these spaces. These experiences are influenced by other people who are present in these spaces [11]. Due to these interactions, “saying inclusion” does not always translate into ‘sensing inclusion’ and eventually ‘doing inclusion’” [52, p. 23].

Our analysis included papers from 1990 to 2023. The use of the term inclusion is not only historically contingent in its meaning but also varies across different socio-cultural contexts and also across disciplines, including law, sport or education. That is making the comparability of inclusive potentials challenging [11]. The expansion of the concept of inclusion, as it was undertaken in the analyzed qualitative studies following the interviews, to include a subjective component and feelings such as belonging, value, and acceptance, demonstrates the necessity of capturing subjective experiences in different spaces. Therefore, more research is needed to investigate these aspects and incorporate them into their conceptual framework [11,52]. A significant amount of the insights into subjective inclusion experiences stems from research in school-based physical education [11,45,54,55]. From our point of view, research in leisure and recreational sports seems equally important, for instance, to assist coaches when designing accessible sports facilities [57] and suitable physical activity programs [11].

In the following, we will address our three sub-questions.

a) How do the authors evaluate the inclusive potential of goalball and wheelchair basketball?

In the analyzed articles, sports were understood as an important tool for inclusion, with the abilities of individuals with disabilities playing a key role in its implementation [27,46,51]. Abilities are seen as a central aspect that goes beyond participation in sports and is crucial for the successful inclusion of people with disabilities. The acquired skills may be significant not only within the context of sports but also in broader societal contexts. Participation in sports fosters the athletes' self-realization. This is in line with previous studies that have shown a positive social identity among wheelchair basketball players [22,50], as well as among blind tennis players [58] and in judoka with intellectual disabilities [59].

Acquiring new skills and improving existing ones leads to a greater sense of autonomy, accomplishment and self-determination. This has been confirmed in studies with young wheelchair users and adult blind tennis players. The athletes used the skills they learned during training to improve their mobility in everyday life [24,58]. Despite these positive aspects, inclusion in these contexts is often defined by ability performances, which can potentially be exclusionary [17]. Holland et al. [10] highlight reasons why individuals may not feel included in integrated settings. Feelings of inadequacy may play a role in this context, which can be traced back to assumed or attributed lack of abilities [60]. “Inclusion language within sport institutions is arguably shaped by normative, ableist structures, which set this specific sports context as the norm or ‘ideal’” [52, p. 26]. In competitive sport events such as the Paralympics with their seemingly objective and fair set of rules, individuals are ranked in relation to performances of abilities considered as relevant which leads to the exclusion of individuals from certain areas of sport activities [17]. Ableism can restrict the participation of people with disabilities in sports if they are not perceived as athletes or if the sport is viewed as rehabilitative rather than competitive [49]. Questions regarding the relationship between inclusion and ability are increasingly being explored in research, but in-depth investigations remain rare [47].

b) What understanding of inclusion do the athletes with disabilities have?

Only limited statements can be made on this question, as none of the studies have explicitly examined this issue. The term inclusion is used in various ways and can refer to both, equal opportunities and a person's sense of belonging. When the term refers to a space, it often remains unclear whether inclusive experiences actually occur within that space [54]. Diverse experiences of people with disabilities are important for capturing, analyzing, and drawing relevant conclusions for sports practice regarding inclusion experiences [52].

c) What is the background of the participants that took part in the studies (gender, age, (dis)abilities)?

The reviewed articles showed a balanced proportion of male and female participants; however, it remains unclear whether this balance is representative for the entire body of research on disability sports. Alhumaid et al. [16] and Carretti et al. [61] point out that gender inequalities in sports, particularly in relation to disability, remain insufficiently investigated. Another research gap refers to the consideration of the experiences of athletes with disabilities at the intersection from other historically marginalized experiences such as LGBTQIA ​+ ​[62], immigration status [63] or refugee experience, whose perspectives are largely absent in the existing studies. Conducting qualitative studies that capture the subjective experiences of people with disabilities in inclusive sports, as suggested by Haegele et al. [12], is of particular importance in this context. This methodological approach is crucial in order to comprehensively understand the complex dimensions of inclusion and the experiences associated with it.

### 4.1 Strengths and limitations

This review represents, to our knowledge, the first of its kind to comprehensively address the topic of inclusion concepts within the context of specific disability sports. The findings significantly contribute to the understanding of the unique challenges and potentials of inclusion in these sports. However, the limited number of studies, particularly in the area of goalball, is a notable limitation. The limited body of research makes it difficult to draw comprehensive conclusions and highlights the need for further investigation in this specific context. In addition, this review only focuses on two specific team sports, which increases the depth of the investigation in these areas, but at the same time ignores the current body of research on disability sports in general. A broader perspective could contribute to a more comprehensive understanding of inclusion in disability sport. In addition, inclusive sport groups outside of disability sport could be investigated to gain deeper insights into the experiences of athletes in different sport programs. Additionally, this review does not take into account other dimensions of diversity such as gender, sexual orientation, and age. Studies conducted by Carretti et al. [61] and Müller and Böhlke [64], demonstrate that these dimensions also play an important role in research on inclusion and should be given more attention. A potential limitation of this study is that the initial screening and selection process was primarily conducted by the first author, with verification by the second author. Future research could enhance methodological rigor by incorporating multiple independent reviewers at all screening stages or by implementing an additional conflict resolution step. Another methodological limitation is the focus on the term ‘inclusion’ in this literature review, while concepts such as ‘integration’ and ‘participation’ were not considered in the screening process. These terms are often used interchangeably in the literature [65], which could result in fewer search results and ambiguity in the interpretation of the findings. This also applies to the language restrictions that have been placed on our search. In order to obtain a more comprehensive overview, future research should employ a more extensive approach. Furthermore, it is generally difficult to find all relevant articles on a particular topic in scientific databases, as the total number of relevant articles is unknown [39]. Therefore, it is possible that not all relevant articles were included in our analysis. In summary, the strength of this review lies in its specific focus and innovative approach. However, it also reveals the need for a broader and more in-depth investigation that systematically incorporates various dimensions of diversity and concepts of inclusion.

## 5 Conclusion

This paper presented findings from a scoping review that examined inclusion concepts in goalball and wheelchair basketball. The novel contribution lies in revealing concepts of inclusion in a disability sport context which has not been investigated before in a scoping review. Important insights were gained regarding both the theoretical and practical significance of inclusion in these sports.

The findings highlight the need for further research employing qualitative methodologies that go beyond the spatial dimension of inclusion, encompassing other dimensions to contribute to a more comprehensive understanding of inclusion in (disability) sport. Moreover, further research should broaden the perspective to other (disability) sports that may have inclusive potential and also focus on leisure and recreational sports when designing suitable physical activity programs, as the discussions towards the concepts of inclusion has been mainly dominated by school-based physical education and activity [4,11]. As ableism can influence sport and physical activity [60] additional research is required to investigate the relationship between inclusion and ability. Future studies should also take into account other dimensions of diversity and how different athletes themselves understand and experience inclusion in sporting contexts with the aim of addressing subjective experiences of inclusion in order to create more inclusive sporting spaces, question ableist structures and language in sporting institutions and design sporting facilities and programs that are accessible to all people.

## Funding

This research received no external funding.

## Declaration of competing interest

The authors declare that they have no known competing financial interests or personal relationships that could have appeared to influence the work reported in this paper.

## Footnotes

1 The terms disability sport and para-sport are often used interchangeably and refer to sports for people with disabilities. In this paper, we use the term disability sport as a broader term that includes a variety of sports and levels of participation.
